# Brain–computer-interface-driven artistic expression: real-time cognitive visualization in the pangolin scales animatronic dress and screen dress

**DOI:** 10.3389/fnhum.2025.1516776

**Published:** 2025-03-06

**Authors:** Leonhard Schreiner, Anouk Wipprecht, Ali Olyanasab, Sebastian Sieghartsleitner, Harald Pretl, Christoph Guger

**Affiliations:** ^1^g.tec Medical Engineering GmbH, Schiedlberg, Austria; ^2^Institute for Integrated Circuits, Johannes Kepler University, Linz, Austria; ^3^Independent Designer, Amsterdam, Netherlands; ^4^Institute of Computational Perception, Johannes Kepler University, Linz, Austria

**Keywords:** BCI, art, uHD EEG, engagement, 3D-print, animatronic, fashion-tech

## Abstract

This paper explores the intersection of brain–computer interfaces (BCIs) and artistic expression, showcasing two innovative projects that merge neuroscience with interactive wearable technology. BCIs, traditionally applied in clinical settings, have expanded into creative domains, enabling real-time monitoring and representation of cognitive states. The first project showcases a low-channel BCI Screen Dress, utilizing a 4-channel electroencephalography (EEG) headband to extract an engagement biomarker. The engagement is visualized through animated eyes on small screens embedded in a 3D-printed dress, which dynamically responds to the wearer’s cognitive state. This system offers an accessible approach to cognitive visualization, leveraging real-time engagement estimation and demonstrating the effectiveness of low-channel BCIs in artistic applications. In contrast, the second project involves an ultra-high-density EEG (uHD EEG) system integrated into an animatronic dress inspired by pangolin scales. The uHD EEG system drives physical movements and lighting, visually and kinetically expressing different EEG frequency bands. Results show that both projects have successfully transformed brain signals into interactive, wearable art, offering a multisensory experience for both wearers and audiences. These projects highlight the vast potential of BCIs beyond traditional clinical applications, extending into fields such as entertainment, fashion, and education. These innovative wearable systems underscore the ability of BCIs to expand the boundaries of creative expression, turning the wearer’s cognitive processes into art. The combination of neuroscience and fashion tech, from simplified EEG headsets to uHD EEG systems, demonstrates the scalability of BCI applications in artistic domains.

## 1 Introduction

Brain-computer interfaces (BCIs) facilitate direct communication between the brain and external devices by translating neural activity into commands or signals, allowing users to control a wide range of functions (Wolpaw et al., 2000). Traditionally, BCIs have been employed in clinical applications, particularly in assistive technologies aimed at restoring communication and mobility for individuals with motor impairments (Birbaumer et al., 2014). These systems allow users to interact with their environment by translating brain signals into meaningful outputs.

Recently, BCIs have expanded beyond clinical settings into broader human-computer interaction (HCI) domains, including entertainment and multimodal interaction. These systems offer communication and control functionalities and enable real-time monitoring of a user’s cognitive and emotional states, allowing environments to adapt dynamically to individuals (Andujar et al., 2015; Nijholt and Nam, 2015; Nijholt, 2019). This advancement illustrates BCIs’ potential to personalize HCI beyond traditional uses, particularly in non-clinical contexts.

One emerging area of BCI research is its integration with artistic expression. BCIs increasingly serve as tools to bridge neuroscience and creative processes. Converting neural signals into visual or physical outputs allows cognitive and emotional states to be dynamically represented in art, enabling real-time interactions between brain activity and artistic expression. This opens novel pathways for exploring human cognition and emotion through art.

Early examples of BCIs in art include Alvin Lucier’s “Music for Solo Performer” (1965), where alpha brainwave rhythms controlled percussion instruments in real-time, pioneering the use of brain signals in live performances (Rosenboom, 1977). Since then, BCIs in art have been categorized into three areas: visualization, where brain signals generate visual or auditory representations of mental states; musification or animation, where neural activity controls artistic tools like animations or music; and instrument control, where brain rhythms manipulate instruments, allowing users to create music or art directly through brain activity (Gürkök and Nijholt, 2013).

BCIs have also redefined the roles of artists and audiences, enabling collaborative art creation. Alfano (2019) demonstrated how BCIs facilitate audience interaction during artistic production, allowing cognitive and emotional states to influence the creative outcome directly. Other artistic BCI systems include those for controlling animations and music (Matthias and Ryan, 2007) or interacting with instruments using brain signals (Muenssinger et al., 2010; Todd et al., 2012).

Eduardo Miranda and colleagues introduced the term Brain-Computer Music Interfaces (BCMIs) to describe BCIs designed explicitly for musical applications (Miranda et al., 2008; Wu et al., 2013). In one example, a BCMI audio mixer allowed users to control the volume of different segments of a pre-composed musical piece by modulating their alpha and beta brainwaves. While users could manipulate the volume, they did not compose the music themselves, making it a selective control system based on brainwave modulation.

Building on this, Chew and Caspary (2011) developed a BCI system for real-time music composition. Utilizing a modified P300-speller interface with an 8×8 matrix, the system provided 64 different note options for users. This allowed users to listen to each note and make subsequent choices, giving them complete control over the composition process.

Yuksel et al. (2015) took this one step further. They introduced a novel advancement in this domain by integrating a BCI with a musical instrument that adapts in real-time to the user’s cognitive workload during improvisation. Unlike previous BCIs, which either map brainwaves to sound or require explicit control, this system implicitly adjusts to cognitive states, using functional near-infrared spectroscopy (fNIRS) to classify workload and modify musical output accordingly. Users reported feeling more creative with this adaptive system compared to traditional approaches.

Furthermore, hyperscanning, a neuroimaging technique that records brain activity from multiple individuals during collaborative tasks, has opened new avenues for exploring neural synchronization and shared cognitive processes in artistic settings (Hasson et al., 2012; Babiloni and Astolfi, 2014; Dikker et al., 2017; Kinreich et al., 2017). This method reveals how multiple brains align during cooperative tasks, providing insights into collective artistic creation.

With the development of more accessible and affordable BCI technologies, artists create interactive installations involving multiple users. These systems often provide real-time feedback, allowing participants to modulate their brain activity to influence the artistic outcomes. This growing trend toward brain-driven, interactive art highlights the potential of BCIs to expand the boundaries of creative expression, offering artists and audiences new and innovative ways to engage with art.

This paper explores two innovative and complementary approaches to merging BCI technology with artistic expression, positioning them within the overarching theme: the artistic representation of brain activity through wearable technology. The dresses were built together with Dutch fashion tech Designer Anouk Wipprecht. While distinct in their technological frameworks, both projects demonstrate the powerful connection between neuroscience and interactive art.

In both projects, we employed Electroencephalography (EEG) for control purposes. EEG acquires brain activity from the scalp’s surface, is easy to use, and has been extensively studied. The outstanding temporal resolution, low price, and convenient usability make EEG the most common method used in BCI research (Mason et al., 2007). Invasive methods such as electrocorticography (ECoG) provide better signal quality. Still, they are impractical for many people due to the need for controlled operating room environments with associated costs and risks and because neurosurgery may not be safe or necessary. Regarding spatial resolution, brain imaging using functional magnetic resonance imaging (fMRI) delivers the best results. However, the comparably lower temporal sampling resolution and the needed space and cost make fMRI unfeasible for many BCI applications. EEG and ECoG systems deliver excellent temporal resolution. In addition, high-frequency oscillations (HFO) or evoked potentials, such as the brainstem auditory evoked potentials (BAEP), that are amongst the fastest evoked potentials, can be acquired using those methods (Chiappa, 1997; Gotman, 2010; Sharifshazileh et al., 2021). However, standard EEG systems provide a comparably low spatial resolution with around 20–60 mm sensor distances. High-density EEG approaches entail more sensors than typical EEG systems and thus can improve spatial resolution.

**Figure 1:**
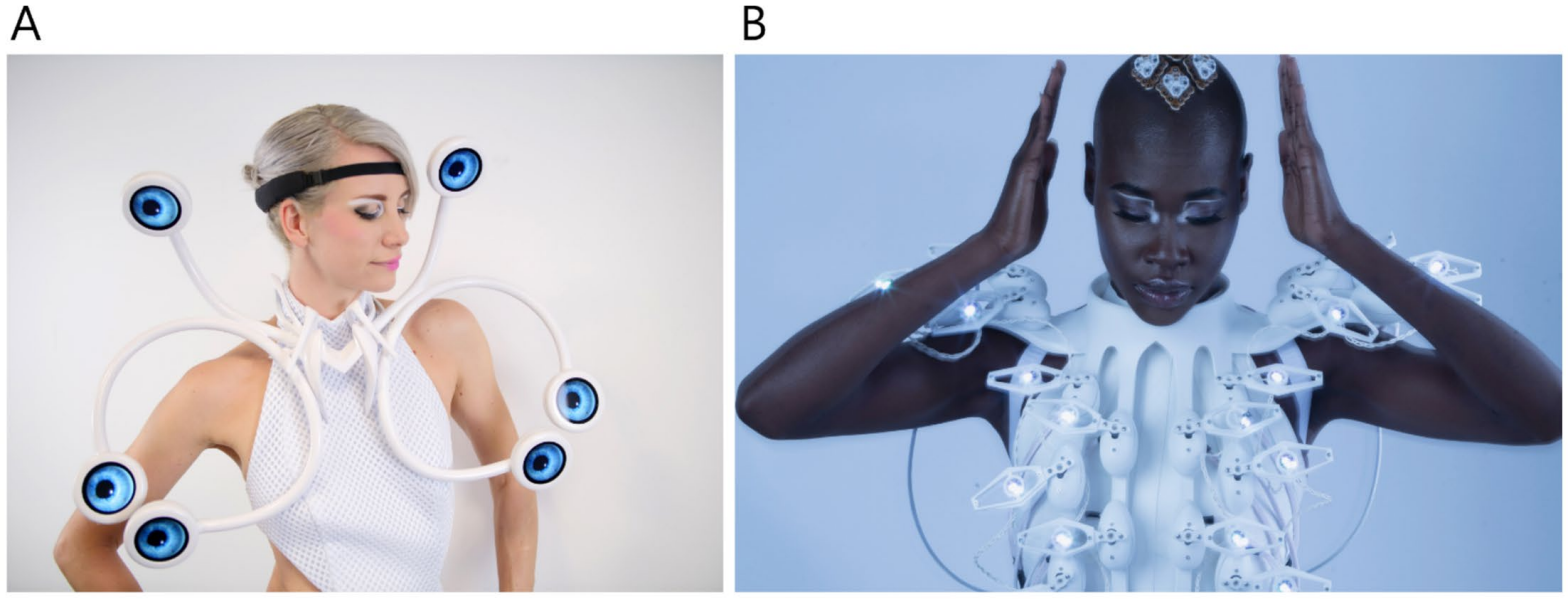
**(A)** Project 1: screen dress (©Anouk Wipprecht), **(B)** Project 2: pangolin scales dress (©Yanni de Melo).

The first project in this paper centers on the low-channel BCI screen dress, a wearable system designed to visualize EEG-based engagement in real-time. Utilizing a 4-channel EEG headband. This system captures the wearer’s engagement and translates it into visual cues. The biomarker applied was based on the study by Natalizio et al. (2024), which focused on real-time estimation of EEG-based engagement across different tasks. In this study, the authors describe the extraction of specific EEG biomarkers to control systems, enabling real-time evaluation of cognitive engagement in various tasks. In our project, digital eyes embedded in the dress screens react to the wearer’s cognitive workload, visually intuitively representing their internal mental processes (see Figure 1A). This low-channel BCI system emphasizes cognitive visualization and accessibility, demonstrating the potential of simplified EEG systems for real-time interaction in artistic and practical applications.

The second project employs an ultra-high-density EEG (uHD EEG) system called g.Pangolin (g.tec medical engineering GmbH), integrated into an animatronic dress inspired by the scales of a pangolin. With 1,024 EEG channels, this system captures high-resolution brain data, which drives the physical movement and lighting of the dress’s animatronic components. The system was applied in research on several topics, including individual finger movement decoding (Lee et al., 2022), hand gesture decoding (Schreiner et al., 2023), and non-invasive mapping of the central sulcus (Schreiner et al., 2024a). The uHD EEG system offers a novel view of non-invasive brain activity. It controls the scales’ movements and lights in response to neural signals, making it a powerful tool for detailed, real-time brain representation. Various EEG frequency bands were visually and kinetically represented in the animatronic dress. Elevated theta power, associated with calm and meditative states, activated slow, steady movements of the scales accompanied by a soft purple glow. Increased alpha power, linked to relaxation and focus, produced a wave-like motion in blue across the dress. Meanwhile, heightened beta power, reflecting alertness and concentration, triggered rapid, mirrored flickering white lights and synchronized scale movements, symbolizing intense cognitive activity (Abhang et al., 2016).

The two brain-computer interfaces build upon and extend previous studies by applying established BCI principles to novel artistic and wearable contexts. The Screen Dress leverages low-channel EEG systems for real-time cognitive visualization, drawing on prior research into EEG-based engagement estimation (e.g., Natalizio et al., 2024) and simplifying the technology for accessibility. The Pangolin Scales Dress integrates ultra-high-density EEG (uHD EEG) technology, building on advancements in high-resolution neural decoding (Lee et al., 2022; Schreiner et al., 2024a, 2024c, 2024d; Schreiner et al., 2024b).

Both projects expand on earlier artistic BCI applications, like music composition and instrument control (e.g., Miranda et al., 2008; Chew and Caspary, 2011), by incorporating real-time visual and kinetic feedback into wearable art. By doing so, these BCIs demonstrate new artistic applications and push the boundaries of interactive BCI technology, connecting neuroscience and art in innovative ways.

The relationship between the two BCI systems in this study is parallel rather than sequential. Both projects— the Screen Dress and the Pangolin Scales Dress—were developed independently to explore distinct yet complementary aspects of BCI-driven wearable art. Together, these two projects explore the potential of BCI technology to create interactive, brain-driven art. One uses cognitive visualization using a low-channel system, and the other uses animatronic responses driven by high-density EEG data. Positioned within the same artistic theme, they reflect different levels of complexity and interaction, demonstrating the broad range of possibilities for artistic representation of brain activity.

The motivation behind these projects stems from the desire to bridge neuroscience, technology, and creative expression, addressing technical and experiential gaps. Traditional approaches in neuroscience and art often fail to engage audiences in an interactive and personalized manner (Nijholt, 2019). These projects utilize BCI technology to translate neural signals into dynamic artistic outputs, enabling real-time visualization of brain activity. By making abstract neural processes tangible, they aim to foster public engagement and explore new paradigms of interaction and creativity. Furthermore, the two projects introduce a new paradigm for interactive and participatory art, allowing users to engage with artistic creations uniquely by dynamically integrating their cognitive states.

## 2 Materials and methods

### 2.1 Project 1: screen dress

#### 2.1.1 BCI technology—screen dress

**Figure 2:**
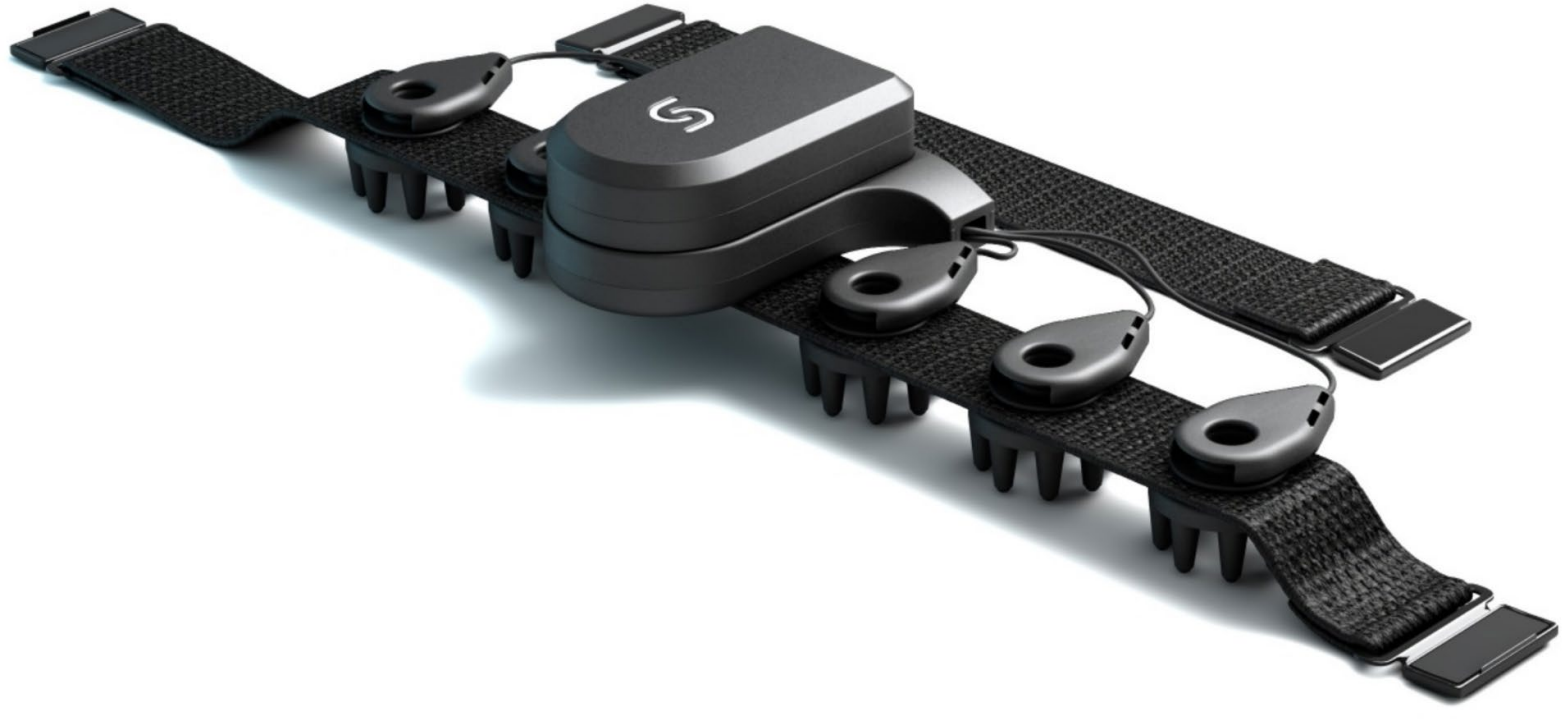
Unicorn BCI Core-4: 4-channel EEG headband device utilizing dry electrode technology.

A novel 4-channel EEG device was developed as part of this project, offering a significant advantage in terms of usability compared to conventional EEG systems. The headband (Figure 2), designed with dry electrodes, is user-friendly and easy to apply, making it accessible to many users. It records four EEG channels from the occipital region, with an additional electrode placed just above the right ear as the reference and ground. The device captures EEG with 24-bit resolution at a sampling rate of 250 Hz. It transmits the data wirelessly via Bluetooth Low Energy (BLE), ensuring efficient and reliable data transmission with minimal latency.

#### 2.1.2 Screen dress

The dress components were designed using PTC’s Onshape cloud-native product development platform (PTC Inc., Boston, MA, USA) and 3D-printed by HP Inc. (HP Inc., Palo Alto, CA, USA) using their Jet Fusion 3D Printing Solution. The dress was fabricated with HP’s HPMulti Jet Fusion 5,420 W printer and HR 3D PA12 W material. Interactive elements of the dress include Hyperpixel 2.1 round displays (Pimoroni Ltd., Sheffield, UK), controlled by a Raspberry Pi Zero 2 W (Raspberry Pi Foundation, Cambridge, UK), featuring a 1GHz quad-core 64-bit Arm Cortex-A53 CPU, 512 MB SDRAM, and 2.4GHz wireless LAN, meeting the system’s visualization requirements. The 3D eyes, designed in Unity (Unity Technologies, San Francisco, CA, USA), were connected to a UDP receiver socket, enabling real-time control of eye dilation and movement based on data received via the network path.

#### 2.1.3 Screen dress—interface

Connecting BCI and the dress allows the dress to react to the wearer’s brain activity in real-time. First, a machine learning algorithm was trained by acquiring data from the specific user in different mental states. Afterward, new data was fed through the BCI. This information was then used in real-time to calculate the level of engagement and visualize it by adapting eye movements on the screens, such as dilation, speed, etc. Data from one representative participant was analyzed and presented in this paper to demonstrate the system’s functionality and performance. The analysis was based on data collected from a 37-year-old healthy female participant during the exhibition settings.

**Figure 3:**
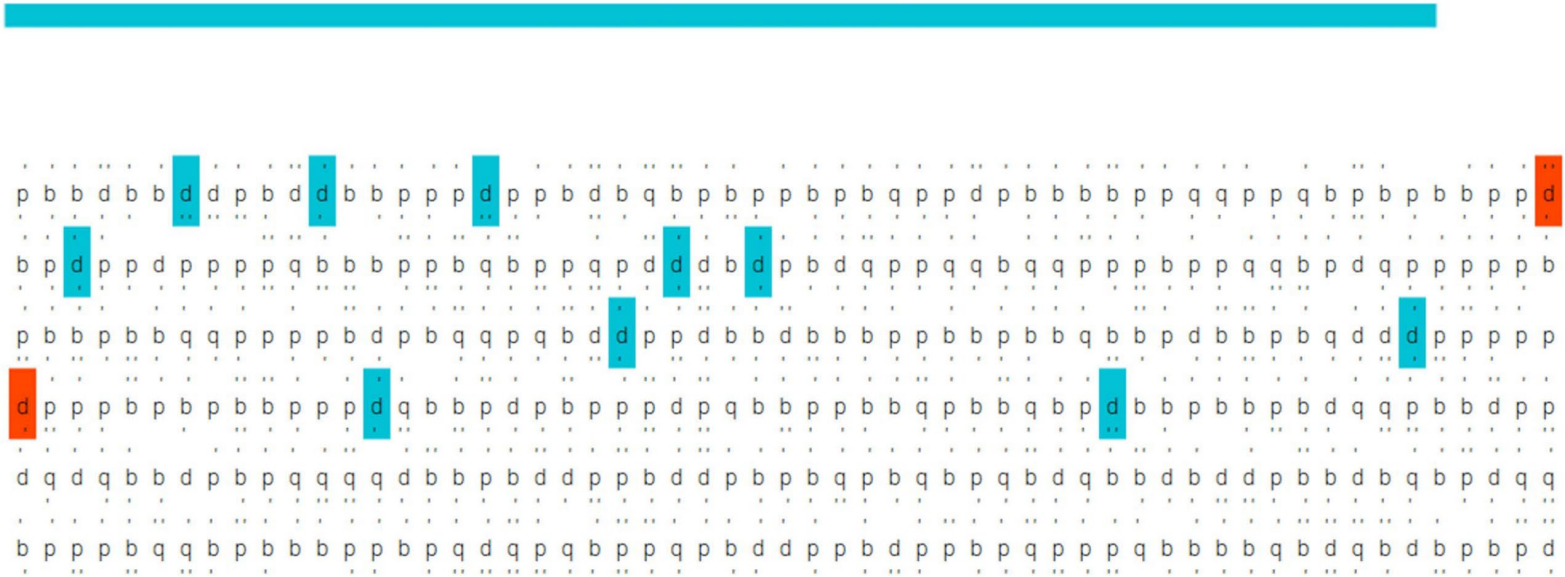
Example of the d2 test performed during the training session [adapted from Natalizio et al., 2024].

As detailed in Natalizio et al. (2024), a specialized application was developed to present stimuli, acquire EEG, and process real-time data. This application processes EEG data to estimate user engagement levels and provides real-time feedback, such as current engagement estimates. For example, in the study by Natalizio et al. (2024), the application involved estimating engagement during gameplay by monitoring participants’ interaction with Tetris at varying speed levels and assessing engagement while watching different video content. Before initiating measurements, the system assesses signal quality, continuously monitoring and reporting noisy channels. Users can also select from various interaction paradigms. Specifically, the d2 test paradigm (Figure 3) was employed to train the classification model, including the d2 test and a fixation cross. The application calculates reliable performance scores based on the d2 test paradigm. After the initial test, the system evaluates the model’s accuracy in distinguishing between engagement (d2 test) and rest (fixation cross) states. The model can be retrained by the user as needed. The calibration phase, including electrode preparation, d2 test execution, and model training, is completed within approximately 5 min. Once trained, the model enables real-time tracking of user engagement in any external task, which, in this project, controls the movement and dilation of 3D-rendered eyes on six hyperpixel screens integrated into the Screen Dress.

The proposed classification model is optimized for low computational cost to support real-time BCI experiments. A filter bank common spatial patterns (CSP) approach was applied to estimate engagement, with models trained individually for each user. Raw EEG signals were notch-filtered at 50 Hz and further processed with bandpass filters (4–8 Hz, 6–10 Hz, and 8–12 Hz) to exclude higher frequency components that could be affected by muscle artifacts from task-related tension. EEG data were segmented into 1-s windows, and CSP was used to extract features that maximize the variance between engagement and resting states. These features were then used to train a linear discriminant analysis (LDA) model, which outputs a binary classification label and a continuous score to estimate user engagement (Natalizio et al., 2024).

**Figure 4:**
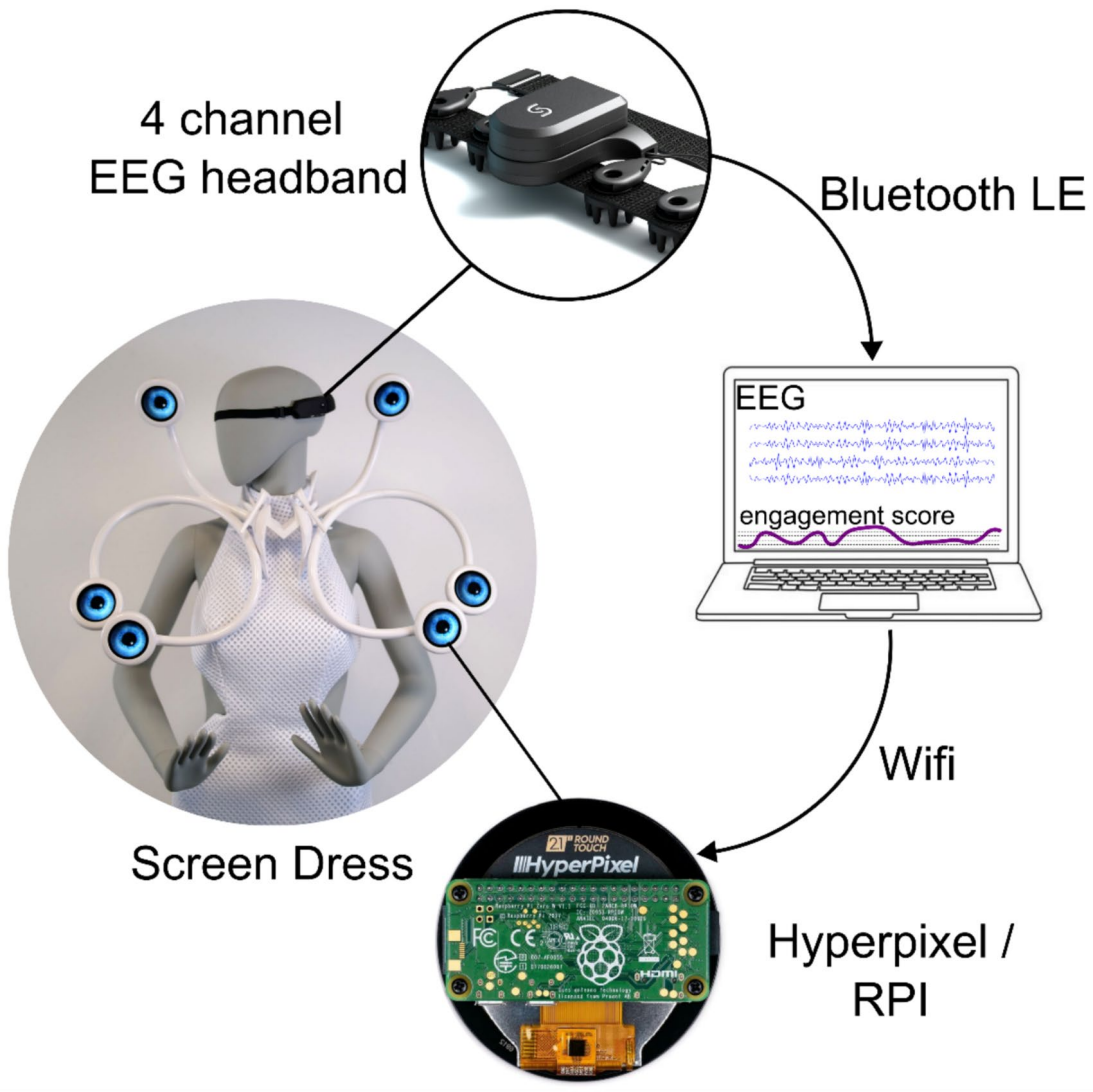
Schematic overview of the system setup: featuring the 4-channel EEG headband for data acquisition, signal processing on PC, and the Hyperpixel for visualization embedded in the Screen Dress.

Figure 4 provides a schematic overview of the BCI control system. The process begins with extracting raw EEG data from the four sensors transmitted via Bluetooth Low Energy (BLE) to the control PC. The PC performs signal processing as described earlier and trains the classifier. After the classifier is trained, the system calculates the scores in real-time and transmits them via Wi-Fi to the Hyperpixel unit. The RPi at the Hyperpixel unit hosts a Unity application that reacts to the values received via UDP and employs them to control eye movements. The scores are divided into two groups: positive and negative. Positive scores, indicating a greater likelihood of class 1 (d2 test), cause the eyes to perform rapid horizontal movements and increase pupil diameter. Negative scores, corresponding to class 2 (resting state), reduce eye movement and pupil constriction. The left and right eye displays mirror each other, following the same movement and dilation patterns.

### 2.2 Project 2: pangolin scales

#### 2.2.1 BCI technology—pangolin scales

##### 2.2.1.1 High-density EEG

Despite a general interest in higher spatial resolution, only a few EEG systems with more than 256 electrode positions covering the whole scalp exist. Since all current systems measuring high-density surface-EEG rely on a cap or a similar stretchable structure to mount the electrodes, the attachment mechanism and the cap limit this approach from becoming spatially denser. Current EEG systems that provide 256 channels often place many electrodes on the cheeks and the neck, which is irrelevant for BCI control (Mammone et al., 2019). The electrode center-to-center distance ranges in such systems between 1 and 2 cm (Luu and Ferree, 2000). Limiting factors for higher densities and wet electrode technologies are bridges between the electrodes and, thus, crosstalk between the channels. Further, poorly defined electrode contact areas can result from using conductive gel or saline electrolyte solutions, limiting the reproducibility of EEG recordings and source localization efforts. Therefore, proper channel differentiation and a consistently low impedance are vital for high-density EEG studies.

##### 2.2.1.2 Ultra-high density EEG

**Figure 5:**
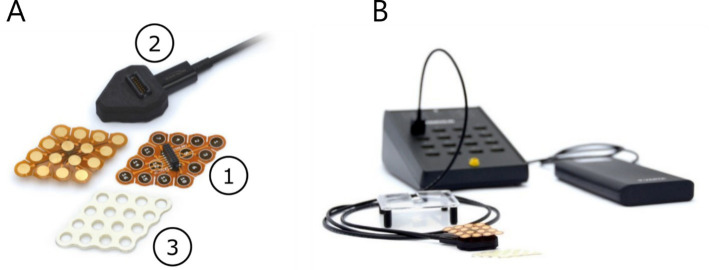
uHD EEG system g.Pangolin. **(A)** electrode grids, pre-amplifier, and medical adhesives, **(B)** connectorbox.

Considering the abovementioned factors, we introduce a novel high-density EEG system, classified as ultra-high-density due to its exceptionally high spatial resolution. The system is called g.Pangolin, inspired by the geometries of the diamond-shaped scales of the pangolin. The electrodes are produced as flexible printed circuit boards (PCB) with gold-plated electrode areas. The diamond-shaped geometry has the excellent property of enveloping the surface of the human skin. For improved deformability on the skull and other body parts, the electrode grid has slits on the sides (see Figure 5A1). The inter-electrode distance is 8.6 mm, with an electrode diameter of 5.9 mm. The adhesive layer is moisture-resistant and insulating medical materials that prevent shortcuts and crosstalk. The holes of the adhesive layer are filled with conductive adhesive paste (Elefix) to ensure optimal skin contact and low impedance at the electrode-skin junction (see Figure 5A3). A pre-amplifier improves the signal quality and has a higher signal-to-noise ratio (SNR). The pre-amplifier is connected to the slim socket connector of the electrode grid (see Figure 5A2). The circuit board amplifies the signals with a fixed gain of 10. A connector box interfaces the high-resolution electrode grids with the pre-amplifier and the biosignal amplifier (see Figure 5B).

**Figure 6:**
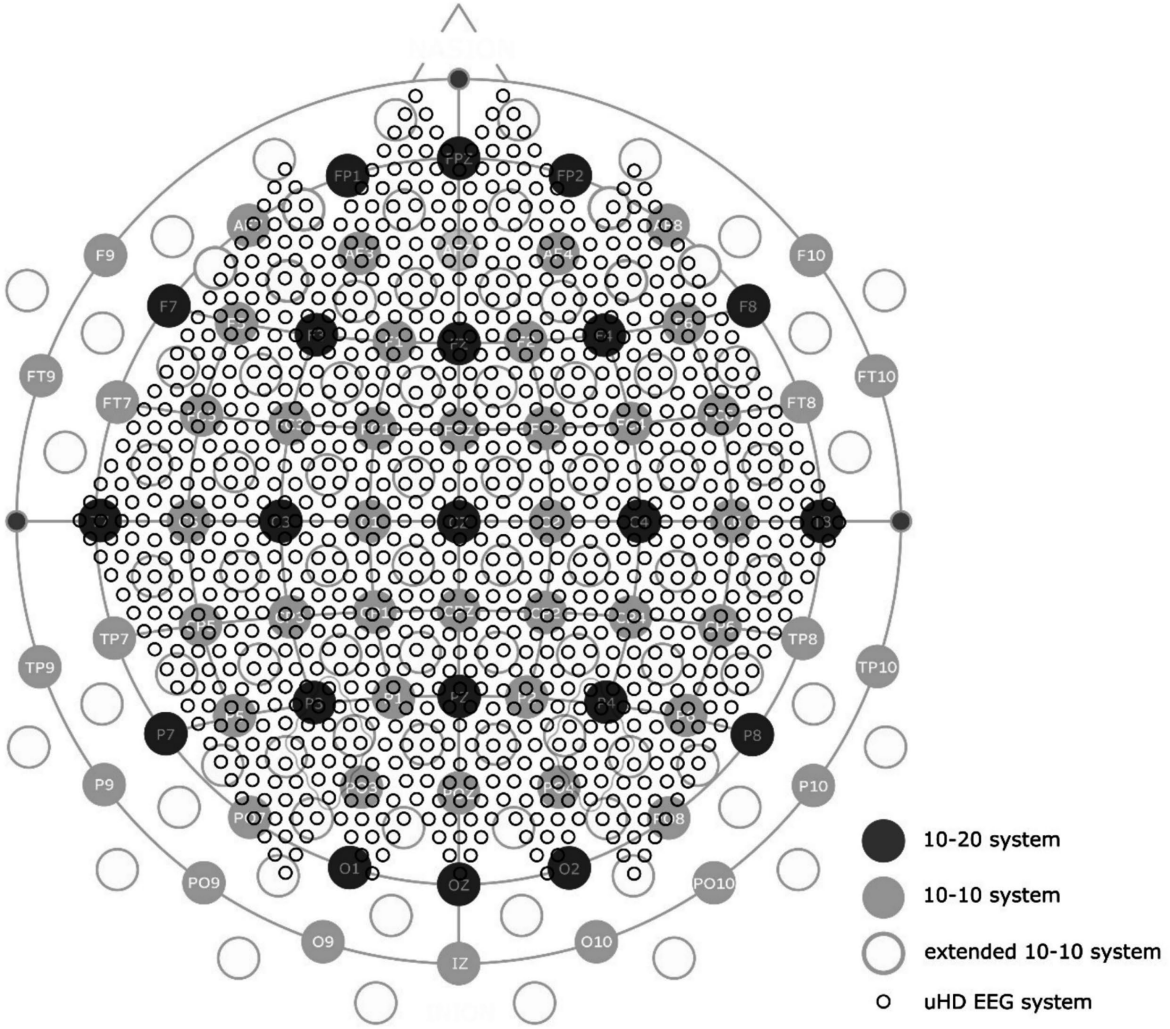
Electrode distribution of the uHD EEG system (small black empty circles) compared to the standard 10-20-system (dark gray filled circles), 10–10 system (light gray filled circles), and the extended 10–10 system (light gray empty circles).

Introduced by Jasper in 1958, the 10–20 system has become state-of-the-art for clinical EEG (Klem et al., 1999). Figure 6 has a general overview of standardized electrode positioning systems, with dark grey circles indicating the 21 standard positions from the 10–20 system. Denser systems, such as the 10–10 system (marked in light grey) and the extended 10–10 system (marked with empty grey circles), can also be seen in Figure 6 (Oostenveld and Praamstra, 2001). The uHD approach has even more positions, indicated by the small empty black circles in Figure 6. Covering the whole scalp using the uHD EEG system results in 1024 electrode positions.

#### 2.2.2 Pangolin scales dress

Figure 1B depicts the Pangolin Dress worn by a model. All the dress parts are 3D printed via selective laser sintering (SLS) from PA-11 and PA-12 (nylon) at Shapeways (Shapeways Inc., New York, NY, USA). This approach led to a very lightweight dress with custom housings for actuators and LEDs mounted on the scales. It also made it possible to design the dress to form something akin to an exoskeleton around the body. With the pangolin as an inspiration, the designer created diamond-shaped scales covering the model’s body. Similar to the ones of the animal that protect the pangolin animal against predators. The housings of the servomotors that move the scales are shaped like eggs. The drive axis led out of the housing so the scales could be mounted. On top of the scales, multicolor LEDs were fixed in a recess.

**Figure 7:**
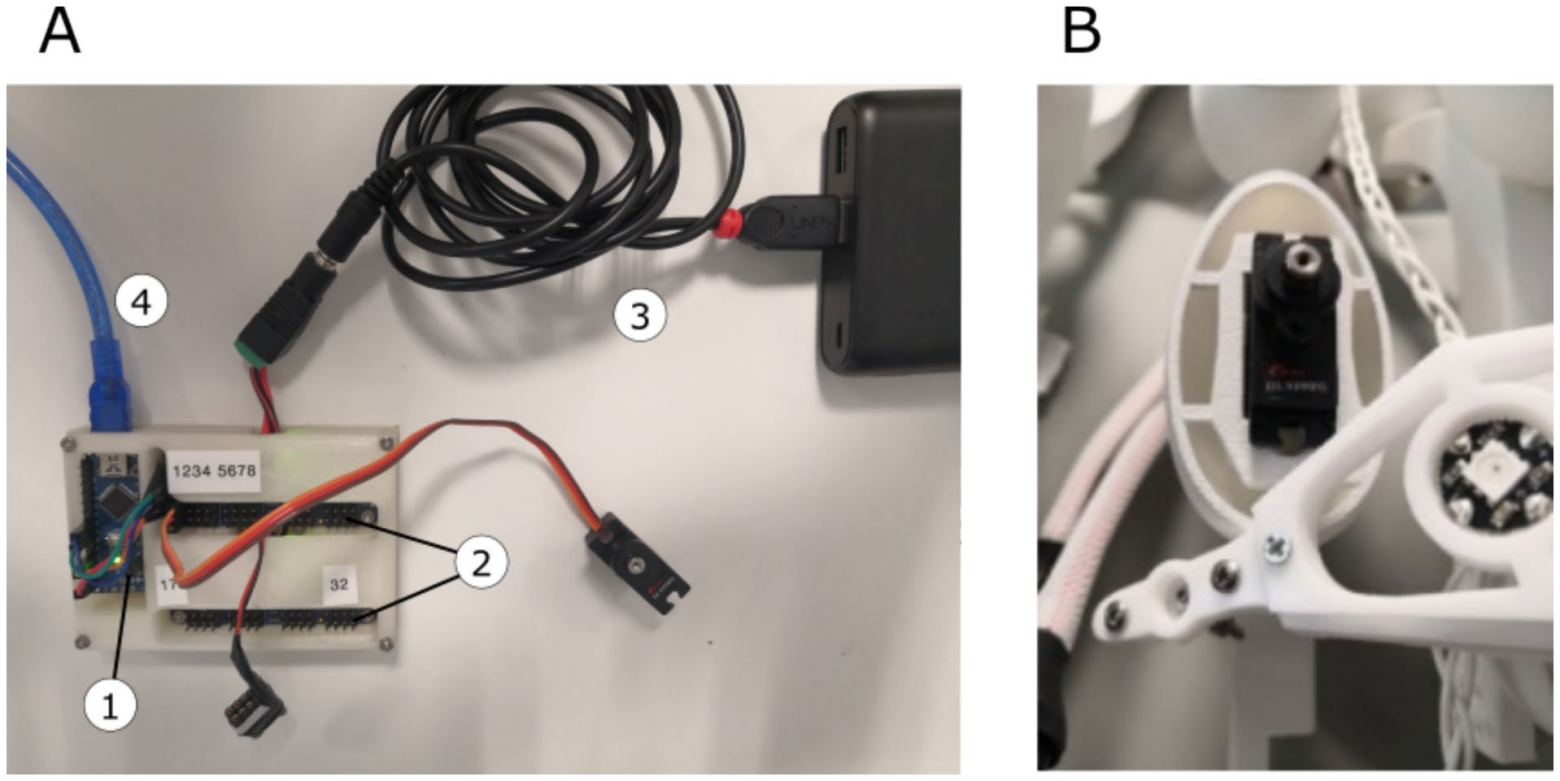
**(A)** Hardware board, including the Arduino nano μC (1) and 2x motor driver boards (2), powered via battery pack (3) and connected via USB to the control PC (4); **(B)** Servo motor and the LED pixel mounted on the dress components.

We used an Arduino nano microcontroller to move the scales and interact with the multicolor LEDs (see Figure 7A1). We chose this board due to its small size, output pin structure, and easy programming interface. Digital metal gear servomotors *(Corona DS-939MG)* were employed for moving the scales. A total of 32 servomotors were installed into the 3D-printed dress. The motors were placed inside the 3D-printed structure and closed via a cover to look like eggs (see Figure 7B). Two (PCA9685) motor driver boards (Adafruit Industries LLC, USA), one of which can drive 16 motors, were adopted for control purposes (see Figure 7A2). Pulse width modulation (PWM) was chosen for interaction with the motors and positioning of the axle. The scale was mounted onto the axle using a distance part for optimal freedom of movement. The entire device was powered by a single battery pack (see Figure 7A3), with data transmitted via USB serial communication (see Figure 7A4).

#### 2.2.3 Pangolin scales—interface

Data from one representative participant was analyzed and presented in this paper to demonstrate the system’s functionality and performance. For this project, the analysis focused on data collected from a 31-year-old healthy female participant during the exhibition settings.

The BCI user in this project was designated as being in an idle state or one of three other states:

1. Theta (*Θ*) – meditation, creativity (dress color purple)

Theta waves (Θ), which range from 4 to 8 Hz, are the slowest frequencies used for control in this BCI system. These waves are commonly associated with states of deep relaxation and inward focus, as well as early sleep stages (Vyazovskiy and Tobler, 2005). Additionally, theta activity in the prefrontal cortex has been connected to the “flow state,” which is characterized by enhanced creativity and cognitive engagement (Katahira et al., 2018). In the system context, the dress color purple represents the presence of theta waves, symbolizing creativity and meditation.

1. Alpha (*α*) – relaxed awake (dress color blue)

Alpha waves (α), ranging between 8 and 12 Hz, are typically observed when users are awake but relaxed, especially with closed eyes. In motor imagery-based BCI research, the alpha band is also called the mu band (Pfurtscheller et al., 2006). Alpha rhythms are most prominent over the occipital cortex at the back of the head and have been linked to various aspects of human life, including sensorimotor functions (Neuper and Pfurtscheller, 2001; Hanslmayr et al., 2005), psycho-emotional markers (Cacioppo, 2004; Allen et al., 2018), and physiological research (Cooray et al., 2011; Halgren et al., 2019). In this system, the dress color blue represents alpha waves, reflecting a state of relaxed wakefulness.

1. Beta (*β*) – alertness, stress (dress color white)

Beta waves (β), which occupy the frequency range of 12–35 Hz, are associated with alertness, attention, and stress. These waves are most prominent when individuals are focused or experiencing stress, making them a key marker of heightened cognitive engagement (Schreiner et al., 2021). Due to the wide range of frequencies, the beta band is often divided into subbands, although precise definitions of these subbands vary in the literature. Frequencies above 35 Hz are classified as gamma waves. They are often discussed in EEG research related to human emotions (Yang et al., 2020) and motor-related functions, mainly through invasive methods such as ECoG (Kapeller et al., 2014; Gruenwald et al., 2017). In the BCI system, white represents beta waves, which signal alertness or stress.

This framework of brainwave frequencies forms the basis of the BCI control system, where changes in cognitive states are mapped to specific visual feedback, as seen through the dynamic color changes of the dress.

**Figure 8:**
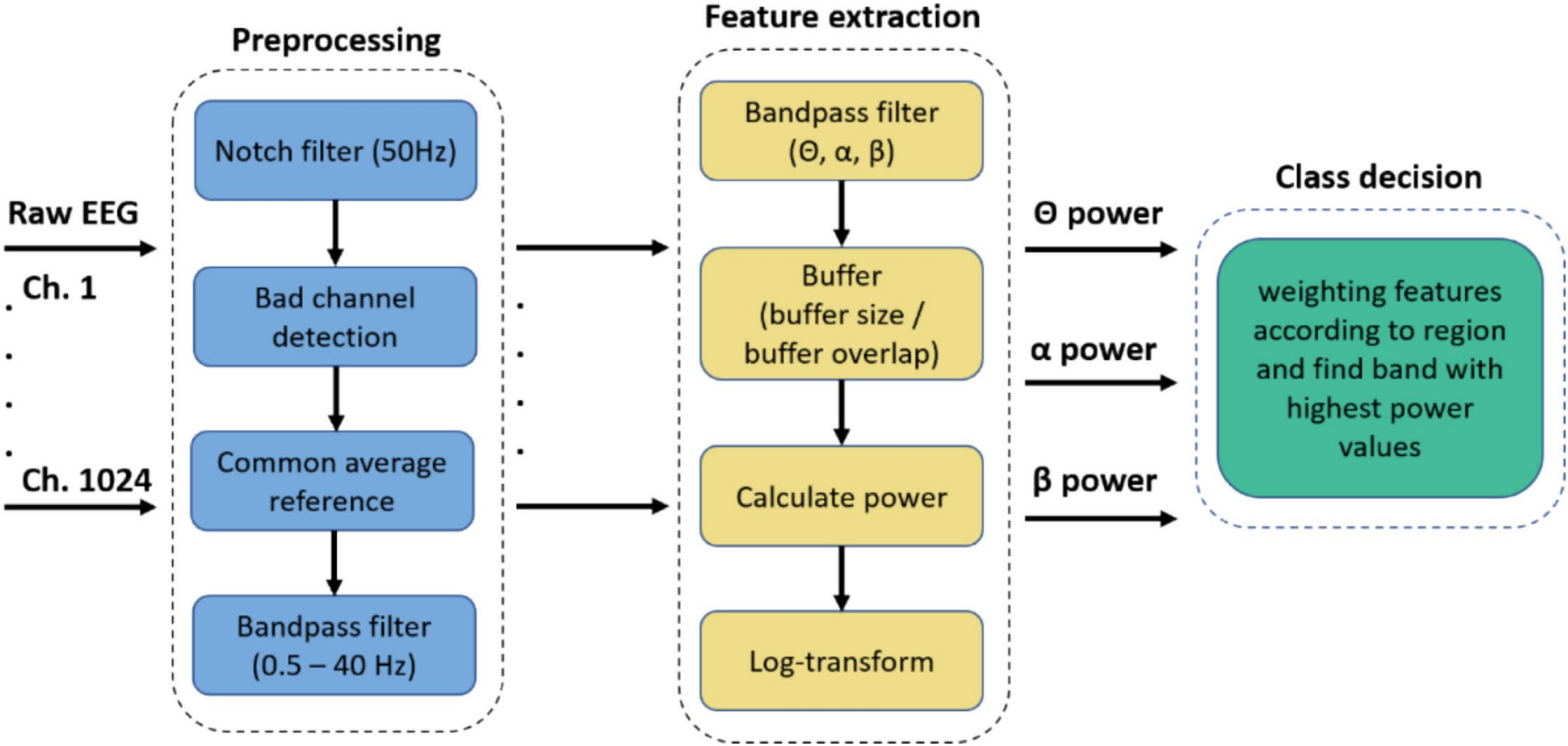
Signal Processing pipeline from the BCI system used for controlling the dress, from preprocessing the raw EEG through feature extraction to the final class decision.

The BCI system must classify features calculated from the measured EEG to enter one of the above states. Data acquisition and online signal processing were performed using g.HIsys Professional (g.tec medical engineering GmbH, Austria) running under MATLAB/Simulink (The MathWorks, Inc., USA). Figure 8 depicts the BCI system’s preprocessing, feature extraction, and class decision steps.

The data were notch-filtered at 50 Hz using a 4th-order Butterworth filter. Next, bad channels were detected using the signal quality scope from the g.HIsys online processing platform. A common average reference (CAR) was used to reference all channels, excluding those classified as having bad signal quality. Finally, a 0.5–40 Hz bandpass filter was selected to pre-filter the frequency band of interest.

For feature extraction, the band powers for the specified frequency ranges were estimated continuously and used for further processing. First, the EEG data were bandpass filtered for the respective frequency band (*Θ*, *α*, *β*). For power estimation, a moving average with a buffer of 256 samples ( $N_{\mathrm{buffer}}$ ) and an overlap of 128 samples ( $N_{\mathrm{overlap}}$ ) was selected. The sampling rate of the EEG amplifier was 256 Hz ( $f_{\mathrm{system}}$ ),which then resulted in an update rate of 2 Hz for the band power features ( $f_{\mathrm{band}}$ ) (see Equation 1) To improve Gaussianity, band power features were log-transformed since they are commonly Chi-squared distributed otherwise. The power from each channel of the uHD EEG systems was calculated online in real-time.


(1) $$f_{\mathrm{band}}=\frac{f_{\mathrm{system}}}{N_{\mathrm{buffer}}-N_{\mathrm{overlap}}}$$

For each channel, the respective frequency band features were calculated (*Θ*, *α*, β). This information was then used in real-time to calculate the dress states’ class decisions.

**Figure 9:**
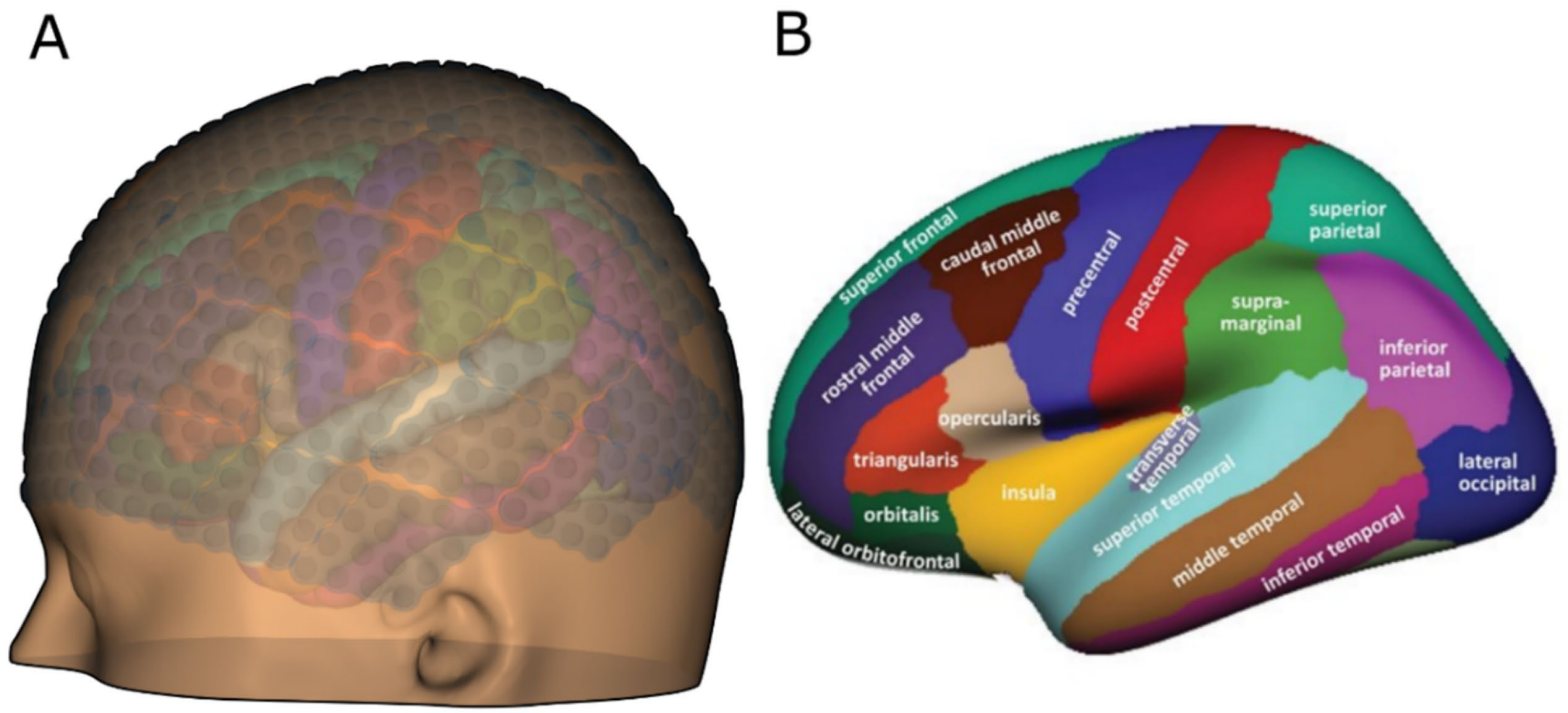
**(A)** Brain model with the uHD grids and functional areas; **(B)** functional brain areas marked according to the Desikan-Killiany atlas as described by Desikan et al. (2006).

To enhance the sensitivity of the BCI system to cognitive states, additional weighting was assigned to the features extracted from electrode grids based on their neuroanatomical locations. These locations were grouped into three key brain regions, as illustrated in Figure 9. Electrode grids positioned over the frontal lobe (green, Figure 9B) were weighted higher for theta wave (Θ) detection, reflecting the region’s role in creative processes and the flow state. The frontal electrodes were specifically assigned a weight of 2 for the theta band, while the weights for the alpha and beta bands remained at 1. The occipital cortex, crucial for visual processing, strongly influences alpha activity. Therefore, electrodes placed over the occipital region (dark blue, Figure 9B) were given a higher weight for alpha waves (α). The occipital region’s weighting for the alpha band was 2, while the beta band remained weighted at 1. Electrodes placed over the pre-and postcentral motor cortices (blue and red, Figure 9B), which are associated with motor control and alertness, were weighted more heavily for beta wave (*β*) detection. These regions were specifically assigned a weight of 2 for the beta band, while the weights for the theta and alpha bands remained at 1.

This weighting strategy ensures that the brain regions most relevant to each state have a stronger influence on the BCI system’s performance.

To assess the system’s performance, we designed specific tasks for each cognitive state (Theta, Alpha, Beta). Each task was performed for 20 trials for 1 min each, providing sufficient data for analysis. The tasks were chosen to elicit targeted brainwave activity associated with the respective cognitive states:

- Theta (Θ): Meditation and creativity

Participants engaged in a guided visualization exercise for 1 min, imagining a calming scenario (e.g., walking on a beach or exploring a forest) while maintaining a meditative state.

- Alpha (α): Relaxed wakefulness

Participants were instructed to sit comfortably, close their eyes, and relax for 1 min without engaging in active thought processes. This condition was selected to encourage alpha activity, which is prominent during relaxed, eyes-closed states.

- Beta (β): Alertness and stress

Participants performed mental arithmetic tasks, such as calculating a series of additions, subtractions, and multiplications (e.g., “573–48 × 2”). This task was designed to induce beta activity associated with cognitive engagement and focus.

## 3 Results

### 3.1 Results project 1: screen dress

#### 3.1.1 BCI interaction screen dress

The BCI system requires calibration for each user. To achieve this, the participant undergoes a training procedure in front of a computer screen. The training protocol consists of two rounds of the d2 test and two resting periods, each lasting 1 min, during which a fixation cross is displayed on the screen. This results in a total training time of 4 min. Following the acquisition of training data, the classifier is trained. A within-subject classification model was developed for the representative subject to distinguish between engaging and resting states using EEG data recorded during a d2 test-based paradigm. The EEG was captured from four electrodes, and the model was trained utilizing filter-bank common spatial patterns and linear discriminant analysis.

**Figure 10:**
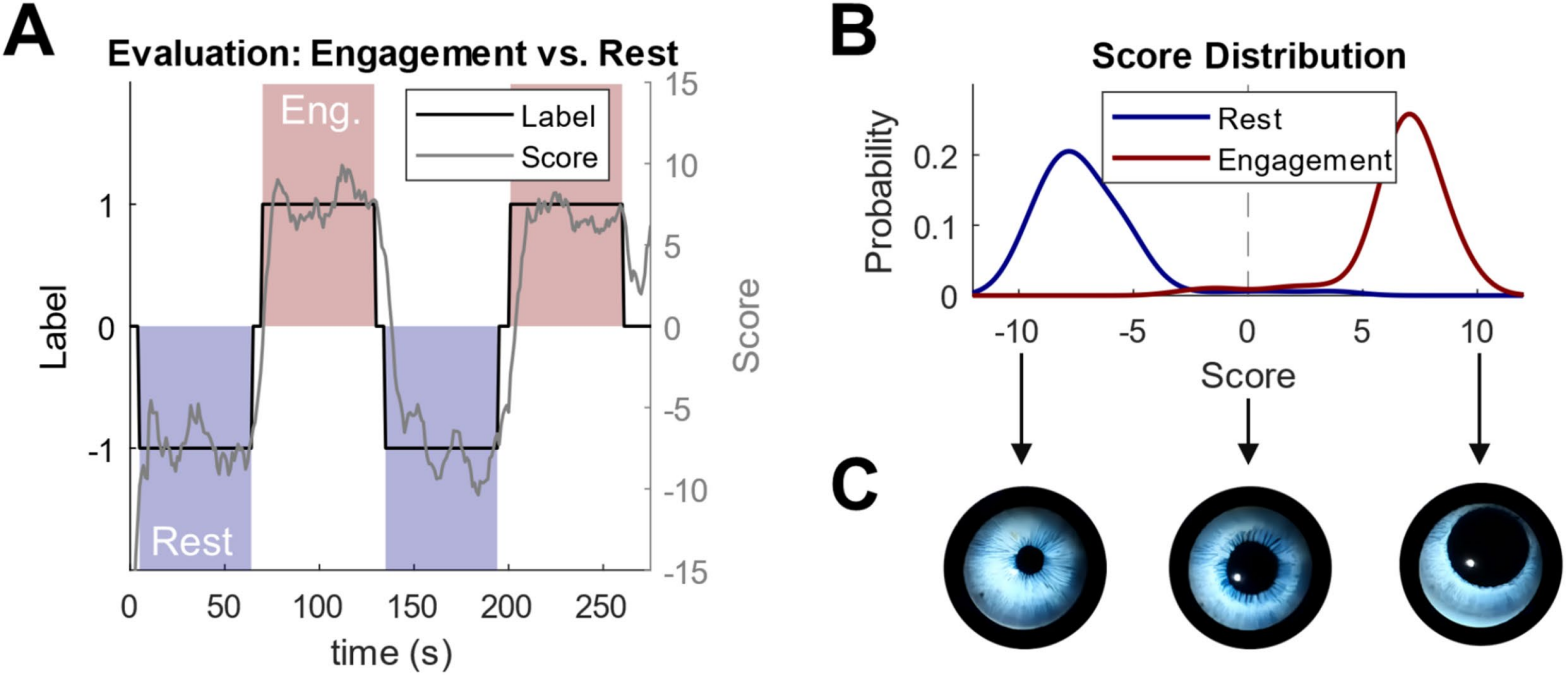
Results of the evaluation run **(A)** Score values over time for engagement condition (red) and rest condition (blue), **(B)** score values for each class, **(C)** 3D-eye animation reaction according to score values and the corresponding condition.

After training, an evaluation run is conducted to assess the classifier’s performance. The calculated scores over time from the evaluation run for the representative subject are shown in Figure 10A. The d2 test, marked in red, is assigned a label of +1, while the resting condition, marked in red, is assigned a label of −1. It is evident that the score values consistently align with the corresponding class for the task being performed. Based on the correct and incorrect estimates during the evaluation run, the classification accuracy reached 97.1%. In contrast, the chance level would be 50% as this is a two-class problem. Furthermore, a significance test using permutation statistics was performed to estimate the probability that the observed performance of 97.1% was obtained by chance (Ojala and Garriga, 2009). Labels and corresponding score values are available in non-overlapping 1-s segments, resulting in 240 segments in total (Figure 10A, 120 s of engaging and 120 s of resting state). The following procedure was performed for B = 10,000 times: Randomly permuting (i.e., shuffling) the labels breaks the relationship between the state and the estimates scores and a permutation accuracy can be calculated, which is based on the assumption that there is no relationship between the labels and the scores. As this procedure was performed 10,000 times, one obtains 10,000 permutation accuracies and then an empirical *p*-value can be computed based on:


(2) $$P=\frac{\#\left({Acc}_{\mathrm{perm}}\geq {Acc}_{obs}\right)+1}{B+1}$$

With ${Acc}_{\mathrm{perm}}$ being the 10,000 permutation accuracies, ${Acc}_{obs}$ being the observed accuracy of 97.1% and B being 10,000 (see Equation 2). In other words, one calculates how often the permutation accuracy was greater or equal to the observed accuracy. Here, we obtained a *p*-value of 9.99E-5 indicating a highly significant model performance.

Figure 10B shows the Score distribution reflects the probability density estimated for the scores during the Rest and Engaging condition, respectively. A non-parametric kernel with a width of 1 was used to fit the distribution.

The trained classifier was applied to new incoming data after calibrating the system and confirming its satisfactory performance. EEG features were extracted in real-time, and corresponding score values were continuously computed and output in real-time. This allowed for constant monitoring and control of the score values. The real-time score was then used to modulate the dilation and movement of the pupil in the animated 3D eye model (see Figure 10C).

To represent the pupil dilation and movement as a function of the score value (ranging from −10 to +10), we can define a linear equation that maps this range to the desired changes in dilation and movement. Let us assume: **s** is the score value (ranging from −10 to +10), *P_dilation(s)* represents the pupil dilation, where a positive score increases dilation, and a negative score decreases it, and *P_movement(s)* represents the pupil movement, which could be proportional to the score value.

We can define the dilation and movement equations as follows:


(3) $$P_{\mathrm{dilation}}\left(s\right)=P0+\alpha *s$$

(4) $$P_{\mathrm{movement}}\left(s\right)=M0+\beta *s$$

Where *P_0* is the baseline pupil dilation (when the score is 0) (see Equation 3), *M_0* is the baseline position of the pupil (when the score is 0) (see Equation 4), α and β scaling factors determine how much pupil dilation and movement change in response to the score. This model assumes a linear relationship for simplicity, but nonlinear models could also be used depending on the desired dynamic behavior.

#### 3.1.2 Exhibition screen dress

The Screen Dress project was showcased at the ARS Electronica Festival 2023^1^. The Ars Electronica Center is a major public science museum in Linz, Austria. Multiple wearers were selected to interact with the Screen Dress during the exhibition using the BCI system. For each wearer, a new classifier was trained and applied to the incoming EEG data streams in real-time. We conversed and interacted with other art exhibits throughout the festival to observe the Screen Dress’s responses. Attendees were notably impressed by the rapid reactions of the digital eyes, which provided subconscious, real-time feedback about the wearer’s engagement. This added an interaction layer, creating a unique experience for bystanders. Videos of the dress, the exhibition, and testing can be found online^2^ (see Supplementary material for corresponding links).

### 3.2 Results project 2: pangolin scales

#### 3.2.1 BCI interaction pangolin scales dress

For each of the 64 electrode grids, the respective frequency band features were calculated in real-time, focusing on theta (*Θ*), alpha (*α*), and beta (*β*) bands. These frequency features were subsequently utilized to determine the state of the animatronic dress. Additional weights were applied to the features based on their corresponding neuroanatomical locations, as shown in Figure 9. The electrode grids were grouped into three distinct neuroanatomical regions for analysis.

Once the frequency features were weighted according to their region, the system performed online classification to decide the dress’s state. The dress could distinguish between three mental states and an idle state. During the idle state, the dress’s motors returned to their default positions, and all LEDs were deactivated.

The system’s performance in detecting cognitive states during task-specific evaluation is summarized in the confusion matrix below. Each cell represents the percentage of trials classified as the predicted state for a given true state. Labels and corresponding accuracy values are computed for non-overlapping 1-s segments. The proportion of total seconds during which power values from specific frequency bands correctly corresponded to the intended cognitive state provides further insight into the system’s accuracy (see Table 1).

**Table 1:** The confusion matrix summarizes the system’s performance in determining cognitive states during task-specific evaluation.

True | Predicted	Theta (Θ)	Alpha (α)	Beta (β)
Theta (Θ)	64%	21%	15%
Alpha (α)	6%	91%	3%
Beta (β)	14%	8%	78%

Rows correspond to the true labels, while columns indicate the predicted labels.

For each state, the observed accuracy (proportion of trials correctly associated with the intended state: Θ: 64%, α: 91%, β: 78%) was compared against a null distribution generated through 10,000 random permutations of the labels (see Equation 1 in BCI Interaction Screen Dress). This approach breaks the relationship between the task and the frequency band power, creating a distribution of accuracies under the assumption of no association. The mean permutation accuracy was 33% confirming the expected chance level under random guessing for a three-state system. The observed accuracies for all three states were significantly higher than the permutation accuracy distribution (*p* < 0.001p).

A minimum interaction time was implemented to ensure a smooth transition between states. The dress was maintained in each state for at least 6 s to avoid rapid changes. Additionally, a threshold for minimum band power was defined to ensure that the BCI system only reacted when the power values in at least one of the frequency bands exceeded this threshold. Once this condition was met, the dress executed the pattern corresponding to the state with the highest power.

After performing the pattern, the system reassessed the frequency power values and, if applicable, updated the dress’s state and corresponding movement and lighting animations. This process allowed the dress to operate autonomously, driven entirely by real-time BCI decisions. However, manual control was also available for demonstration purposes.

The dress looks angelic when it is turned off (idle). In action, it becomes a flowing canvas of color, express and admirable in form and movement. The dress scales move up and light up with animated color patterns depending on the wearer’s brain state. The dress remained in one of the four states until it changed to a new state or was turned off. The following patterns were programmed:

##### 3.2.1.1 Theta (purple)

**Figure 11:**
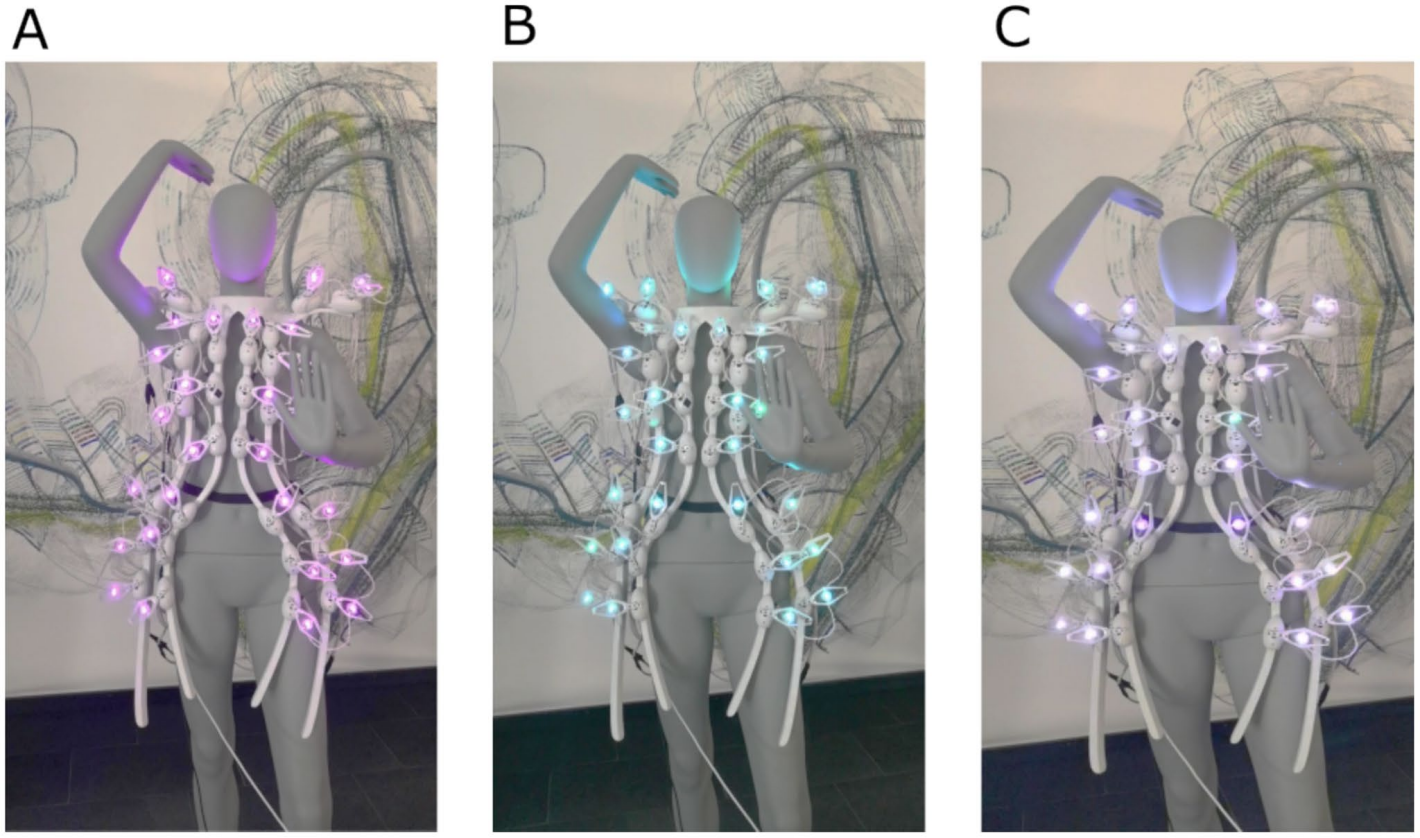
The three dress states with the dress mounted on a mannequin. **(A)** Theta–meditation, creativity (purple), **(B)** Alpha–relaxed, awake (blue), **(C)** Beta–alertness, stress (white).

The theta state should represent calm and meditative behavior. The LEDs glowed slowly in purple, and the scales moved steadily and slowly (see Figure 11A). The movement pattern started at the bottom of the dress and spread through it. When the movement reached the servos at the shoulders, the pattern repeated in the opposite direction.

##### 3.2.1.2 Alpha (blue)

Since the alpha state is supposed to represent a relaxed and focused attitude, the dress should act accordingly. To achieve this effect, an imitation of a wave was designed to pass through the dress by activating the scales sequentially, beginning from one lower end of the dress. The movement spread throughout the dress and ended at the opposite lower end. The LEDs were activated simultaneously to a moving scale to increase the intensity of the movement (see Figure 11B).

##### 3.2.1.3 Beta (flickering white)

We chose a hectic movement pattern for the Beta state to reflect focus and alertness. Hence, the scales quickly moved up and down. Specifically, the scales moved up promptly from their starting positions to around 60 degrees, then returned to the starting position at the same speed after around 0.5 s. The left and right dress sides were mirrored to establish the desired effect (see Figure 11C).

##### 3.2.1.4 Idle (off)

The dress returned to its starting position. All LEDs turned off.

#### 3.2.2 Exhibition pangolin scales dress

**Figure 12:**
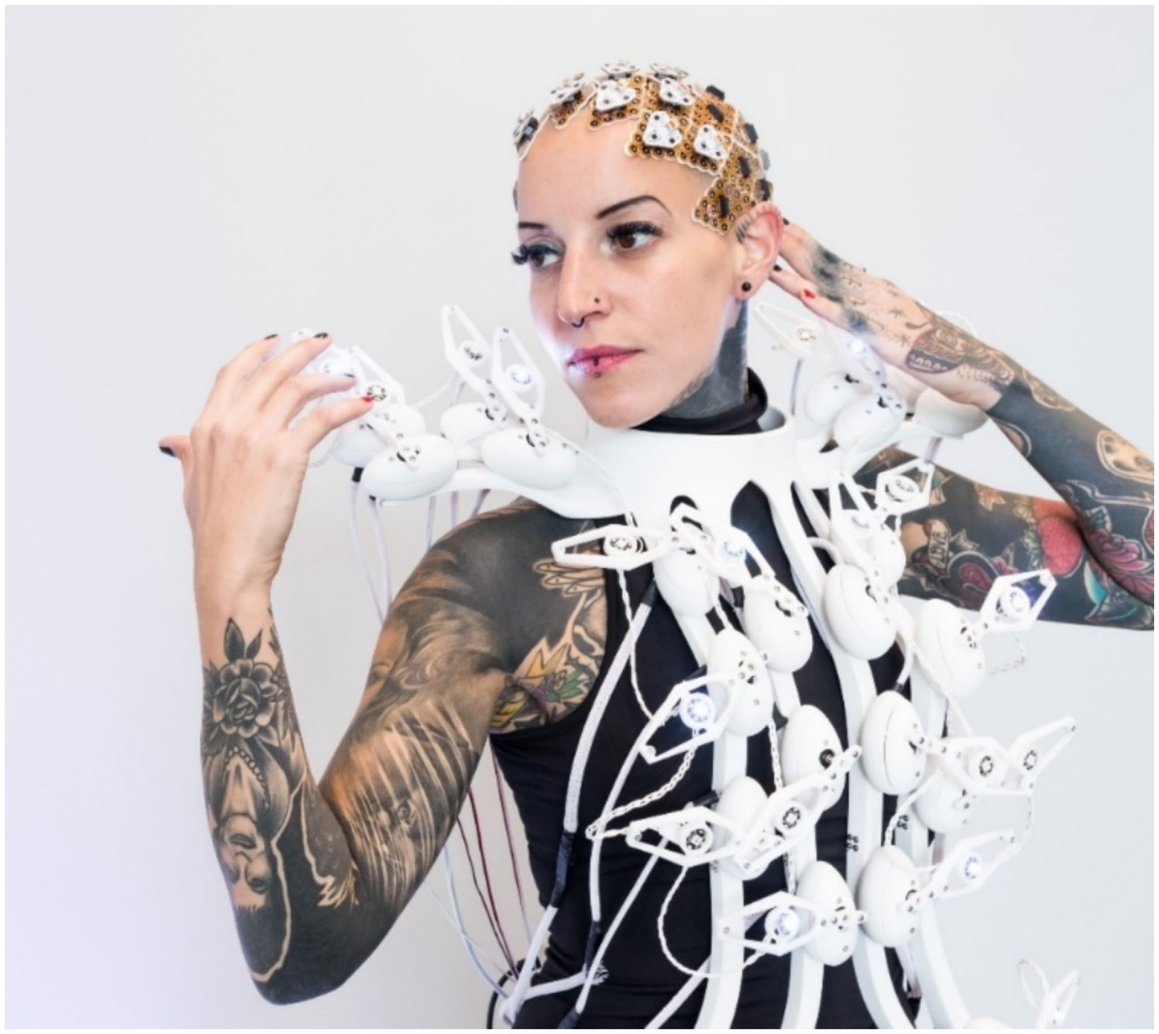
Pangolin Scales EEG electrode grids and interactive dress worn by the model (©Florian Voggeneder).

We presented the pangolin scales project at the ARS Electronica Festival 2020^3^. A model wore both the BCI and the dress for this presentation. The live presentation included preparing the BCI system (electrode preparation, mounting, data acquisition procedure) and the actuation of the dress. Finally, both components were linked, and the dress performed its animations according to the model’s brain state determined by the BCI. The positioning of all 64 electrode grids (preparing them and attaching them to the scalp) took about 2 h (see Figure 12). We asked the model wearing the Pangolin dress to actively engage and interact with the venue, allowing the dress to respond in real-time to the model’s cognitive states. This dynamic feedback provided an additional interactive layer, offering bystanders a unique and immersive experience. Videos documenting the dress in action and footage from the exhibition and testing are available online^4^ (see Supplementary material for corresponding links).

## 4 Discussion

Integrating Brain-Computer Interfaces into wearable artistic projects, such as the Screen Dress and the Pangolin Scales Dress, demonstrates the evolving intersection of neuroscience and creative expression. These projects highlight how real-time neural data can enhance interactivity and audience engagement in novel and meaningful ways.

In the Screen Dress project, a low-channel EEG system (4 channels) monitors cognitive engagement. Visual cues, such as dynamic digital eyes, reflect the wearer’s neural activity in real-time. This approach emphasizes accessibility, utilizing simplified EEG to provide direct feedback on the wearer’s mental state. By using digital eyes to display engagement, this wearable tech makes an individual’s cognitive processes visible, blending fashion with a functional, brain-driven interface. The simplicity and biomarker extraction capabilities of the BCI can be applied across various fields, such as gaming, education, and training. For example, in gaming, the paper by Natalizio et al. (2024) showcased the application of these biomarkers in playing Tetris. This technology could be used in education to assess classroom engagement, similar to the hyper-scanning approach mentioned by Dikker et al. (2017). Another potential application could be in workplace environments, where BCI technology could be used to monitor employee engagement and optimize work planning and break schedules, as demonstrated by the work of Lu et al. (2020) and Wang et al. (2023), as well as in virtual reality (VR) environments to enhance user experience and interaction (Souza and Naves, 2021).

Conversely, the Pangolin Scales Animatronic Dress employs a more complex uHD EEG system with 1,024 channels. This allows for precisely capturing neural signals that drive physical movements and lighting changes in response to cognitive states. Each frequency band (Theta, Alpha, Beta) triggers different visual and mechanical outputs, creating a rich, kinetic representation of the wearer’s brain activity. This high-resolution system showcases the potential of BCIs in generating detailed, real-time artistic representations of brain functions aside from ongoing research (Lee et al., 2022; Schreiner et al., 2023, 2024a) in a creative manner. The new uHD EEG technology is an advanced way to understand the brain and its functions. The uHD EEG described in this chapter may improve several application fields relative to standard EEG systems. The system has already shown its capabilities in the medical area, especially for pre-operative localization purposes. Another field of interest is to have a more precise picture of the seizure onset zones in patients with epilepsy. Further, detecting individual finger movements, which is not yet possible with standard EEG, would be a significant step in BCI research that can be achieved with this system. Experiments on decoding single-finger movements using the uHD system were performed by Lee et al. (2022).

The guided visualization task elicited Theta (*Θ*) activity with a classification accuracy of 64%. Misclassifications occurred primarily as Alpha (*α*) (21%) due to overlap with relaxation states and as Beta (*β*) (15%) during moments of increased mental focus or distraction. The eyes-closed relaxation task demonstrated the highest classification accuracy at 91% for Alpha (α), with minimal misclassifications (6% as Theta and 3% as Beta). The mental arithmetic task achieved 78% accuracy for Beta (β) classification. Misclassifications included 14% as Theta. The results indicate that the system reliably detected the targeted cognitive states for the designed tasks, with performance well above chance levels. This highlights the robustness of the system in identifying brainwave activity associated with specific mental states during task-specific evaluation. However, the impact of the weighting approach should be carefully considered when interpreting these outcomes.

The projects also have broader implications for the fields of art, fashion, and human-computer interaction. By incorporating BCIs into wearable art, these projects open new avenues for exploring the relationship between technology, the brain, and artistic expression. They demonstrate that BCIs are not limited to clinical or research applications but can also be powerful tools for personal and creative expression.

In fashion, these projects challenge traditional notions of clothing as purely aesthetic or functional objects. Instead, the dresses become extensions of the self, reflecting the brain’s inner workings in real-time. This approach could revolutionize the fashion industry by introducing a new category of brain-driven wearables, allowing individuals to express their mental and emotional states through clothing.

In the broader field of human-computer interaction, these projects highlight the potential of BCIs to create more personalized and adaptive systems. By using real-time brain data to control external devices, BCIs could be used to create interactive environments that respond to the user’s cognitive and emotional states. This could have applications in art and fashion and entertainment, education, and therapy, where adaptive environments could enhance user experiences and outcomes.

The Screen Dress and Pangolin Scales Animatronic Dress represent pioneering steps in integrating BCIs with wearable technology for artistic expression. The Screen Dress offers an accessible, real-time cognitive visualization platform using low-channel EEG. At the same time, the Pangolin Scales Dress showcases the potential of uHD EEG to create intricate, kinetic representations of brain activity. Both projects blur the lines between neuroscience, technology, and art, offering new ways to engage with and represent the brain’s inner workings.

Both projects have limitations, including the analysis of data from only one representative participant, which limits the generalizability of the findings. The uHD EEG system requires extensive preparation time and shaved hair for optimal signal quality, making it less practical for broader applications. In contrast, the four-channel EEG headband offers ease of use but suffers from limited spatial resolution, reducing its sensitivity to specific brain regions. Expanding participant diversity and improving system practicality are key areas for future work.

However, these projects not only push the boundaries of what is possible with wearable technology but also redefine the role of the artist and audience in the creative process. By allowing the brain to drive real-time artistic expression, these wearables offer a deeply personal and interactive form of self-expression, opening up new possibilities for the future of brain-driven art and fashion. As BCIs evolve, their potential to revolutionize artistic expression and human-computer interaction will only grow, offering exciting opportunities for future innovations.

## Data Availability

The raw data supporting the conclusions of this article will be made available by the authors, without undue reservation.

## Footnotes

^1^ https://ars.electronica.art/who-owns-the-truth/en/screen-dress/

^2^Video of the dress: https://www.youtube.com/watch?v=FP7SNdYF3Y0.

^3^ https://ars.electronica.art/keplersgardens/de/the-pangolin-scales/

^4^Video of the dress: https://www.youtube.com/watch?v=KSiF5seJnbc&t=15s.
