# Supplementary material for: Brain–computer-interface-driven artistic expression: real-time cognitive visualization in the pangolin scales animatronic dress and screen dress
Source: Front Hum Neurosci. 2025 Mar 6;19:1516776. doi: 10.3389/fnhum.2025.1516776 (PMC11925262; doi:10.3389/fnhum.2025.1516776)
Supplement: Supplementary file 1 [file Data_Sheet_1.pdf]

Supplementary Material

1 Supplementary Data

1.1 Screen Dress - Public available information:

https://www.fastcompany.com/90947968/this-dress-reads-your-mind-so-your-coworkers-dont-have-to

https://www.voxelmatters.com/anouk-wipprecht-releases-mind-controlled-3d-printed-screendress/

https://www.designboom.com/technology/anouk-wipprecht-screendress-3d-printed-dress-eyes-brain-sensor-09-07-2023/

https://www.hackster.io/news/a-dress-that-speaks-louder-than-words-ac02505d7449

1.2 Pangolin Scales Dress - Public available information:

https://www.fastcompany.com/90545032/this-stunning-dress-can-read-minds

https://www.voxelmatters.com/pangolin-scales-bcidress-project-adds-neurotechnology-to-a-3d-printed-dress/

https://spectrum.ieee.org/the-tech-behind-a-mind-reading-dress-could-lead-to-wireless-batteryless-exoskeleton-control

https://www.hackster.io/news/the-pangolin-dress-can-read-your-mind-and-visualize-what-you-re-thinking-c9298ebfb5f6
